# A Novel Fault Diagnosis Scheme for Rolling Bearing Based on Convex Optimization in Synchroextracting Chirplet Transform

**DOI:** 10.3390/s20102813

**Published:** 2020-05-15

**Authors:** Guanghui You, Yong Lv, Yefeng Jiang, Cancan Yi

**Affiliations:** 1 Key Laboratory of Metallurgical Equipment and Control Technology, Ministry of Education, Wuhan University of Science and Technology, Wuhan 430081, China; youguanghui@zime.zj.cn (G.Y.); lvyong@wust.edu.cn (Y.L.); 2 Hubei Key Laboratory of Mechanical Transmission and Manufacturing Engineering, Wuhan University of Science and Technology, Wuhan 430081, China; 3 Zhejiang Institute of Mechanical & Electrical Engineering, Hangzhou 310053, China; 4 Jiaxing Institute of Special Equipment Inspection, Jiaxing 314050, China; jxjyf197902@163.com

**Keywords:** synchroextracting chirplet transform, convex optimization, time-frequency analysis, fault diagnosis

## Abstract

Synchroextracting transform (SET) developed from synchrosqueezing transform (SST) is a novel time-frequency (TF) analysis method. Its concentrated TF spectrum is obtained by applying a synchroextracting operator into TF transformation co-efficients on the TF plane. For this class of post-processing TF analysis methods, the main research focuses on the accurate estimation of instantaneous frequency (IF). However, the performance of TF analysis is greatly affected by the strong frequency modulation (FM) signal. In particular, the actual measured mechanical vibration signals always contain strong background noise, which decreases the resolution of TF representation, resulting in an inaccurate ridge extraction. To solve this problem, an improved penalty function based on the convex optimization scheme is firstly introduced for signal denoising. Based on the superiority of the linear chirplet transform (LCT) in dealing with modulated signals, the synchroextracting chirplet transform (SECT) is employed to sharpen the TF representation after the convex optimization denoising operation. To verify the effectiveness of the proposed method, the numerical simulation signals and the measured fault signals of rolling bearing are carried out, respectively. The results demonstrate that the proposed method leads to a better solution in rolling bearing fault feature extraction.

## 1. Introduction

As one of the most important components in rotating machines, the stable and regular running of rolling bearing can ensure the reliability of whole mechanical equipment. Therefore, it is of great significance to realize the structural health monitoring of rolling bearing [1,2]. Due to the complexity of the mechanical transmission chain, the measured vibration signals of rolling bearing always present strong non-stationary characteristics [3,4]. However, the traditional frequency-domain analysis method based on a fast Fourier transform (FFT) can only be utilized to process stationary signals. Therefore, the joint time-frequency analysis approach, which can reveal the time-varying properties of a signal, has been greatly studied by researchers [5,6,7].

Commonly, the collected vibration signal is a typical non-stationary time series in engineering practice. More characteristic information can be obtained if the signal is transformed to the time-frequency (TF) domain. Undoubtedly, the TF transform is an important technique for non-stationary signal processing. TF transformation is the representation of a one-dimensional time-domain signal on a two-dimensional TF plane, obtaining a two-dimensional function of signal energy changing with time and frequency. The short-time Fourier transform (STFT) [8] is a common TF analysis method based on the Fourier transform during a short window. However, the window function with fixed width often causes the TF resolution of the signal to decline seriously. The wavelet transform (WT) [9] was proposed based on the inner production between the analyzed signal and the wavelet basis function. Nevertheless, the performance of WT is heavily dependent on the choice of the wavelet basis function [10]. Recently, adaptive signal decomposition algorithms, such as the empirical mode decomposition (EMD) and variational mode decomposition (VMD), have been widely used in fault diagnosis [11,12]. However, the actual analysis results are often unsatisfactory due to the lack of theoretical foundation and the difficulty of optimal parameter selection.

The rearrangement method (RM) is a typical post-processing technique that can effectively improve TF resolution [13]. It is characterized by the concentration of TF energy near the instant frequency or the interested frequency of the analyzed signal. Therefore, the TF energy of the rearranged spectrum is more concentrated and has better TF resolution than that of the original TF plane. Unfortunately, the RM algorithm does not support signal reconstruction. In order to overcome the deficiency of traditional spectral rearrangement techniques, I. Daubechies et al. [14] accurately estimated the instant frequency by phase spectrum. Further, a synchrosqueezing operator can obtain a more precise TF spectrum as well as perfect signal reconstruction, which is referred to as the synchrosqueezing transformation (SST) [15]. Nevertheless, the synchrosqueezing operator of SST only considers the frequency coefficient along scale direction. When dealing with a strong frequency modulation (FM) signal, the ideal TF representations can be hard to obtain due to a larger energy divergence in the original TF spectrum. Thus, SST is just applied for simple harmonic signal processing. To analyze frequency modulation signals with different characteristics, demodulation and the iterative demodulation approach have been researched [16]. Since the analyzed phase function is unknown, it is obvious that this method is not suitable for practical application. Then, the high-order synchrosqueezing transform (HSST) was proposed for multi-component signal analysis [17]. However, the computation cost has also increased due to the high-order approximation of the phase function. Based on this, the synchroextracting transform (SET) has been presented [18,19]. The significant difference is that the original synchrosqueezing operator is replaced by a synchroextracting operator. It cannot be denied that SET has an obvious advantage in enhancing the TF energy concentration, but its identified TF trajectories may deviate from the true instantaneous frequencies (IFs) in dealing with strong frequency modulation modes. Based on the above study, Zhu proposed a novel TF analysis method called the synchroextracting chirplet transform (SECT) [20], which combines the characteristics of a synchroextracting operator and a linear chirplet transform (LCT). As a generalized transform of STFT and WT, LCT has many excellent properties with a time-varying window function[21,22]. Although the TF aggregation of SECT has greatly progressed from SET, its TF representation performance is still influenced by noise interference.

When the SECT method considers the reassignment in the instantaneous frequency direction, the TF transformation coefficients of both the useful signals and noise components simultaneously exist in the TF plane. Therefore, inevitable noise components will lead to poor noise robustness of the SECT method. In order to overcome this problem, a novel TF analysis method based on the convex optimization algorithm is put forward to gain a concentrated TF representation of a fault feature curve in this paper. In conventional convex optimization approaches, $l_{1}$-norm is widely applied as a penalty term to obtain the sparse approximate solution for linear equations. However, the $l_{1}$-norm will underestimate the sparsity of true solutions, and it is ineffective in dealing with strong time-varying signals [23,24]. Thus, a novel penalty function named the generalized mini-max concave (GMC) penalty is presented in this paper, which is aimed at substituting the $l_{1}$-norm [25,26]. Compared with *l*_1_ norm regularization, the GMC penalty can provide a more accurate estimation of high-amplitude components of sparse approximation solutions. In this way, the accuracy of sparse solutions has been greatly enhanced. After the noisy vibration signal is disposed by the GMC penalty, the precise TF representations can be identified by the SECT method, and the proposed method in this paper is called the improved SECT (ISECT). In order to verify the performance of the proposed method ISECT, a numerical simulation signal analysis and a feature extraction to measure the rolling bearing vibration signal with outer ring fault have been carried out, respectively.

The rest of this paper is arranged as follows: In Section 2, we give a theoretical introduction to convex optimization based on the GMC penalty and the synchroextracting chirplet transform (SECT). In Section 3, the frequency modulation signal with added Gaussian white noise is presented to verify the effectiveness of the proposed ISECT method. In addition, the results acquired by STFT, SST, and SET are employed to make a comparison. In Section 4, the measured rolling bearing fault signals from the experiment rig and the industrial site are used to further demonstrate the capacity of ISECT. Conclusions are given in Section 5.

## 2. Theory Description

### 2.1. Convex Optimization Based on GMC Penalty

To obtain a more accurate approximation solution of the raw signal, a non-convex penalty function called the generalized mini-max concave (GMC) penalty is employed to substitute the traditional regularization term $l_{1}$-norm in a convex optimization problem.

Firstly, we define the sparse approximate solution to the original signal. The specific solution process can be interpreted by minimizing the least squares cost function:

(1) $$G(x)=\frac{1}{2}{\left\|y-Ax\right\|}_{2}^{2}+\lambda {\left\|x\right\|}_{1}$$

where $y\in R^{M}$ is the raw signal, ${\left\|x\right\|}_{1}$ denotes the $l_{1}$-norm operator to ***x***, $A\in R^{M\times N}$ is an over-sampled inverse discrete short-time Fourier transform operator, and λ>0 corresponds to the regularization parameter. We implement the STFT as a normalized tight frame, i.e., $AA^{H}=I$.

Theoretically, *l*_0_ norm is difficult to be optimized (NP problem), and *l*_1_ norm is the optimal convex approximation of *l*_0_ norm. It also should be pointed out that *l*_1_ norm is easier to be optimized than *l*_0_ norm. However, this may underestimate the true solution. Some studies have shown that a non-convex penalty function as a regularization term can maintain the convexity of the cost function. In this paper, we introduce the non-convex penalty function GMC as the regularization [25], which is defined as a generalization of the Huber function and the mini-max concave penalty function.

The Huber function is firstly defined as:

(2) $$s(x)=\left\{\begin{array}{l} \frac{1}{2}x^{2},\;\;\;\;\;\;\;\;\;\left|x\right|\leq 1\\ \left|x\right|-\frac{1}{2},\;\;\;\left|x\right|\geq 1 \end{array}\right.$$

Then, the mini-max concave penalty function ϕ(x) can be expressed as:

(3) $$\phi (x)=\left\{\begin{array}{l} \left|x\right|-\frac{1}{2}x^{2},\;\;\left|x\right|\leq 1\\ \frac{1}{2},\;\;\;\;\;\;\;\;\;\;\;\;\;\;\;\left|x\right|\geq 1\;\;\; \end{array}\right.$$

According to Equations (2) and (3), we obtain the mini-max concave penalty function ϕ(x) as follows:

(4) $$\phi (x)=\left|x\right|-s(x)$$

where s(x) is the Huber function, and $\left|x\right|$ represents the absolute value of x.

Subsequently, a scaling version of the Huber function $s_{b}$ is proposed for the multi-variate generalization of ϕ(x):

(5) $$s_{b}(x)=\left\{\begin{array}{l} \frac{1}{2}b^{2}x^{2},\;\;\;\;\;\;\;\;\;\left|x\right|\leq \frac{1}{b^{2}}\\ \left|x\right|-\frac{1}{2b^{2}},\;\;\;\;\;\left|x\right|\geq \frac{1}{b^{2}} \end{array}\right.$$

where b≠0. From Equation (5), the corresponding scaled MC penalty is described as:

(6) $$\phi_{b}=\left\{\begin{array}{l} \left|x\right|-\frac{1}{2}b^{2}x^{2},\left|x\right|\leq 1/b^{2}\\ \frac{1}{2b^{2}},\;\;\;\;\;\;\;\;\;\;\;\;\;\;\left|x\right|\geq 1/b^{2} \end{array}\right.$$

Similar to Equation (4), the scaling penalty function $\phi_{b}(x)$ can be denoted as:

(7) $$\phi_{{}_{b}}(x)=\left|x\right|-s_{b}$$

where $s_{b}$ is the scaling MC penalty function.

Then, let $B\in R^{M\times N}$, and we can thus define the generalized Huber function $S_{B}$:

(8) $$S_{B}(X)=\underset{v\in R^{N}}{inf}({\left\|v\right\|}_{1}+\frac{1}{2}{\left\|B(x-v)\right\|}_{2}^{2})$$

By generalizing the $l_{1}$-norm and the Huber function, the generalized mini-max concave penalty function can be expressed as:

(9) $$\psi_{B}(x)={\left\|x\right\|}_{1}-S_{B}$$

where $S_{B}$ is the generalized Huber function and ${\left\|x\right\|}_{1}$ represents the $l_{1}$-norm of x.

Ultimately, Equation (1) can be rewritten as:

(10) $$F(x)=\frac{1}{2}{\left\|y-Ax\right\|}_{2}^{2}+\lambda \psi_{B}(x)$$

where $\psi_{B}$ is the generalized mini-max concave penalty function, which is parameterized by matrix B. Additionally, the selection of matrix B significantly depends on A. In order to maintain the convexity of the above objective function defined in Equation (10), the convexity condition is presented as follows:

(11) $$B^{T}B\leq \frac{1}{\lambda }A^{T}A$$

When the inverse short-time Fourier transform matrix ***A*** is given, Equation (11) can be illustrated as $B=\sqrt{\gamma /\lambda }\;A,\;\;0\leq \gamma \leq 1$. It is obvious that the penalty function is equivalent to the $l_{1}$-norm when we let γ=0. In the experiment, the optimal range of γ can be chosen as 0.5≤γ≤0.8.

To address the problem of minimization in Equation (10), we need to transform it into a typical saddle-point problem:

(12) $$\left(x^{opt},v^{opt})=\arg\;\underset{x\in R^{N}}{\min}\;\underset{v\in R^{N}}{\max}\;F(x,v\right)$$

where $F(x,v)=\frac{1}{2}{\left\|y-Ax\right\|}_{2}^{2}+\lambda {\left\|x\right\|}_{1}-\lambda {\left\|v\right\|}_{1}-\frac{\gamma }{2}{\left\|A(x-v)\right\|}_{2}^{2}$ is the saddle function, and $x^{opt}$ is the optimized result. Furthermore, we can solve the minimization problem by the forward-backward (FB) algorithm [27].

### 2.2. Synchroextracting Chirplet Transform

The chirplet transform (CT) is generalized from the wavelet transform (WT) and has many excellent properties of the short-time Fourier transform (STFT), which is defined as

(13) $$C_{f}^{g}(t,\omega ,\beta )=\int_{-\infty }^{+\infty }f(\mu )g(\mu -t)e^{-\frac{j\beta {\left(\mu -t\right)}^{2}}{2}}e^{-j\omega (\mu -t)}d\mu$$

where f(t) is the analyzed signal, g(t) is a real and even window function in the Schwartz class, and β is the chirplet ratio (CR) parameter.

Based on the CT model, a new rotating parameter α is introduced:

(14) $$\beta =\frac{F_{s}}{2T_{s}}\tan(\alpha )$$

where $T_{s}$ corresponds to the sampling time, and $F_{s}$ is the sampling frequency. We assume the parameter α can be described as:

(15) $$\alpha_{k}=-\frac{\pi }{2}+\frac{k\pi }{N_{\alpha }+1}k=1,2,\ldots ,N_{\alpha }$$

Based on the former description, we can rewrite the expression of a chirplet transform as:

(16) $${C^{\prime }}_{f}^{g}(t,\omega ,\beta )=\int_{-\infty }^{+\infty }f(\mu )g(\mu -t)e^{-j\frac{F_{s}}{2T_{s}}\frac{\tan(\alpha_{k}){\left(\mu -t\right)}^{2}}{2}}e^{-j\omega (\mu -t)}d\mu$$

Theoretically, CT has a satisfied TF representation around its IF with concentrated energy. Moreover, the amplitude $\left|{C^{\prime }}_{f}^{g}(t,\omega ,\beta )\right|$ can reach the maximum among all values. Thus, the best optimization solution of α from the amplitude of $\left|{C^{\prime }}_{f}^{g}(t,\omega ,\beta )\right|$ can be expressed as:

(17) $$\alpha^{*}=\arg\max\left|{C^{\prime }}_{f}^{g}(t,\omega ,\beta )\right|$$

Then, the matching CT $C_{f}^{g}(t,\omega ,\beta )$ can be calculated as

(18) $$C_{f}^{g}(t,\omega ,\beta )=\left\{\begin{array}{l} {C^{\prime }}_{f}^{g}(t,\omega ,\beta )\;\;\;\;\;\;if\left|{C^{\prime }}_{f}^{g}(t,\omega ,\alpha^{*})\right|>\lambda \\ 0\;\;\;\;\;\;\;\;\;\;\;\;\;\;\;\;\;\;\;\;\;\;\;\;otherwise \end{array}\right.$$

Later, the quantity $\tilde{\omega }(t,w)$ is divided by:

(19) $$\tilde{\omega }(t,w)=ℑ\left\{\frac{\frac{\partial }{\partial t}{C^{\prime }}_{f}^{g}(t,\omega ,\beta )}{{C^{\prime }}_{f}^{g}(t,\omega ,\beta )}+\beta^{*}\frac{\frac{\partial }{\partial \omega }{C^{\prime }}_{f}^{g}(t,\omega ,\beta )}{{C^{\prime }}_{f}^{g}(t,\omega ,\beta )}\right\}$$

where $\left|{C^{\prime }}_{f}^{g}(t,\omega ,\alpha^{*})\right|>\lambda$ and $\beta^{*}=\frac{F_{s}}{2T_{s}}\tan(\alpha^{*})$. Therefore, a novel TF representation called the synchroextracting chirplet transform (SECT) [20] is proposed as:

(20) $$Tc(t,\omega )=C_{f}^{g}(t,\omega )\delta (\omega -\tilde{\omega }(t,w))$$

It should be pointed out that the $\delta (\omega -\tilde{\omega }(t,w))$ is a synchroextracting operator, and it can be expressed as:

(21) $$\delta (\omega -\tilde{\omega }(t,w))=\left\{\begin{array}{l} 1\;\;\;\;\;\;\;\left|ℑ\left\{\frac{\frac{\partial }{\partial t}{C^{\prime }}_{f}^{g}(t,\omega ,\beta )}{{C^{\prime }}_{f}^{g}(t,\omega ,\beta )}\right\}\right|<\frac{\Delta \omega }{2}\\ 0\;\;\;\;\;\;\;\;otherwise \end{array}\right.$$

**Figure 1:**
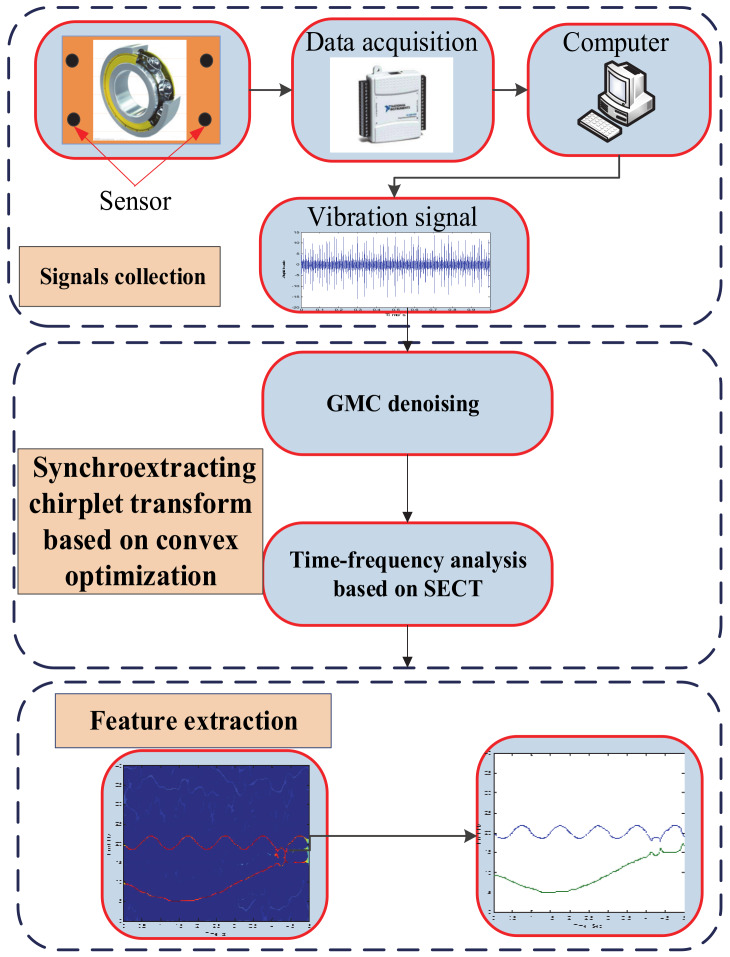
The flowchart of the proposed method.

To achieve the high-resolution representations of a multi-component signal, the proposed method ISECT has combined the advantage of a convex optimization scheme based on the GMC penalty and the TF analysis method of SECT. The specific process of the approach proposed in this paper is shown in Figure 1.

## 3. Simulated Signal Analysis

**Figure 2:**
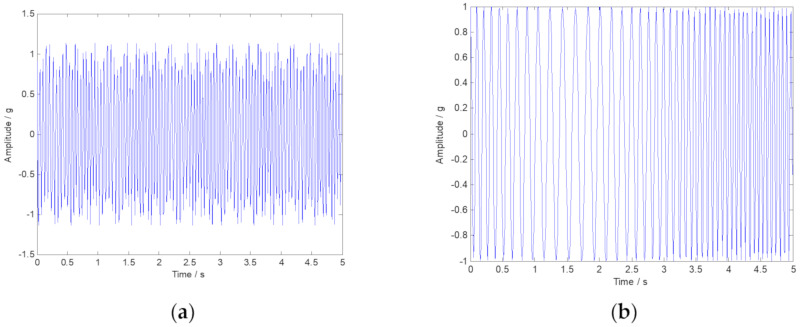
Time-domain waveform of signals *x*_1_ and *x*_2_. (**a**) The amplitude modulation (AM)-frequency modulation (FM) signal *x*_1_; (**b**) The FM signal *x*_2_.

**Figure 3:**
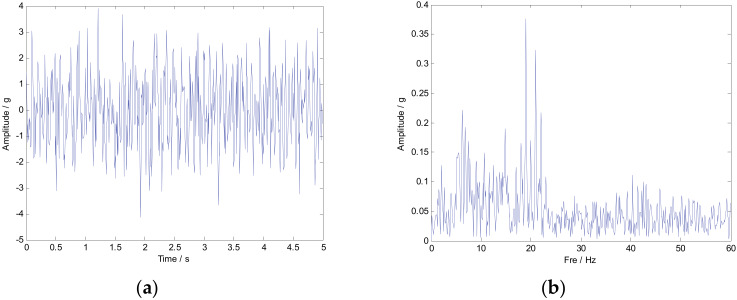
The multi-component signal shown in time and frequency domains. (**a**) Simulated signal shown in time-domain; (**b**) Simulated signal shown in frequency-domain.

To verify the effectiveness of the proposed method, a simulated signal analysis is performed firstly. Commonly, mechanical equipment vibration signals consist of amplitude modulation (AM) and frequency modulation (FM) signals under strong background noise [28]. Thus, the simulated signal is defined as:

(22) $$x_{1}=(1+0.2\sin(2\pi f_{1}t))\sin(2\pi f_{2}t+2\cos(2\pi f_{3}t))$$

(23) $$x_{2}=\cos(2\pi (10t+5\cos(t)))$$

(24) $$x=x_{1}+x_{2}+n$$

where the simulated signal ***x*** is composed of three parts: ***x***_1_, ***x***_2_, and ***n***. Obviously, ***x***_1_ is a typical AM-FM signal, and ***x***_2_ is a FM signal. The characteristic frequency is defined as $f_{1}=15$ Hz, $f_{2}=20$ Hz, and $f_{3}=1$ Hz. It should be noted that the ideal instantaneous frequency of $x_{1}$ and $x_{2}$ can be calculated as $IF_{1}=20-2\sin(2\pi t)$ and $IF_{2}=10-5\sin(t)$, respectively. The symbol ***n*** represents Gaussian noise components with SNR=5 dB. Figure 2 shows the time-domain waveform of signals $x_{1}$ and $x_{2}$. The multi-component numerical simulation signal is displayed in Figure 3.

**Figure 4:**
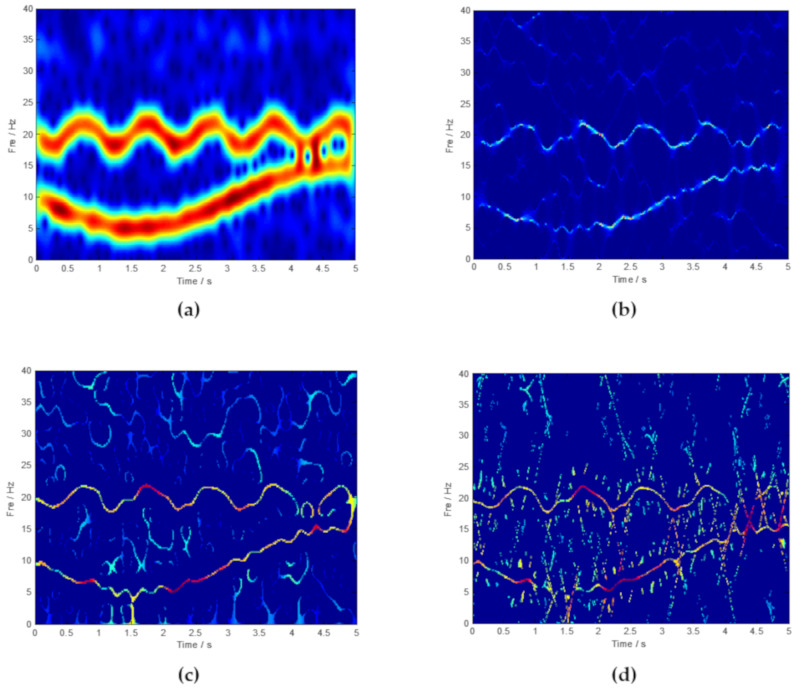
The results of time-frequency (TF) representations provided by (**a**) short-time Fourier transform (STFT), (**b**) synchrosqueezing transformation (SST), (**c**) synchroextracting transform (SET), and (**d**) synchroextracting chirplet transform (SECT).

It is obvious that the traditional signal processing methods such as FFT cannot be effective in identifying time-varying features such as IF_1_ and IF_2_. The main reason is that FFT lacks the ability to analyze non-stationary and aperiodic signals. Subsequently, mainstream time-frequency analysis methods, such as STFT, SST, SET, and SECT, are used to analyze the simulated signal. The TF representations results are shown in Figure 4.

**Figure 5:**
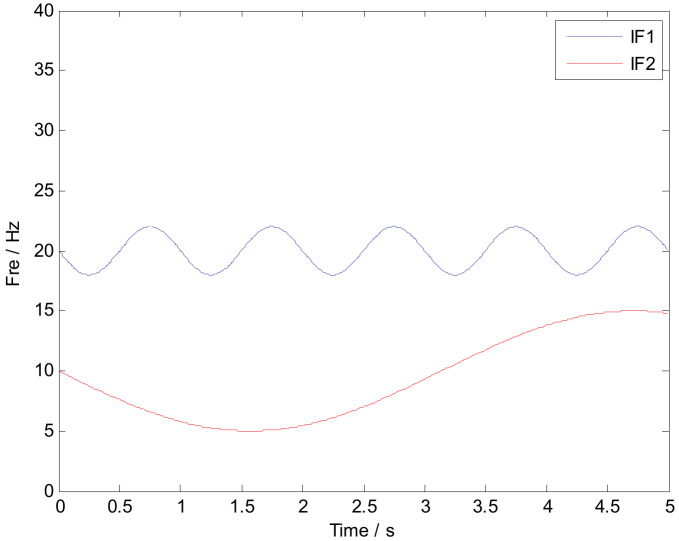
The ideal instantaneous frequency corresponding to IF_1_ and IF_2_.

According to Figure 4, a traditional time-frequency analysis method like STFT has a poor ability in achieving concentrated TF representation. Compared with STFT, popular methods such as SST, SET, and SECT have obvious superiority in a TF analysis. To some extent, the ridge corresponding to the AM-FM signal *x*_1_ and the FM signal *x*_2_ can be inspected. Theoretically, based on the chirplet transform and the synchroextracting operator, SECT has better analysis results. SET and SECT are based on the mathematical foundations of SST. When the instantaneous frequencies of the interested components are squeezed and rearranged, the noise components are still distributed on the time-frequency plane. Thus, the calculated performance of the above-mentioned methods should be improved due to the existing noise components. The most effective solution is to first reduce the noisy components and then optimize the time-frequency representations. Figure 5 is the ideal time-frequency curve corresponding to *x*_1_ and *x*_2_.

**Figure 6:**
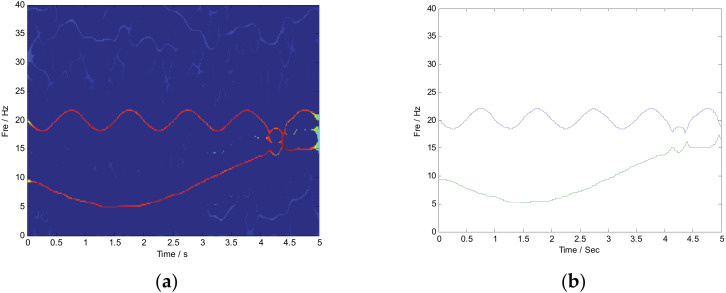
The result calculated by the proposed improved synchroextracting chirplet transform (ISECT) method. (**a**) Result provided by the proposed method; (**b**) Extracted ridge form the optimized TF plane.

According to the previous analysis, we can make a conclusion that the noise components have a negative effect in sharpening the TF ridges. Thus, the GMC denoising approach is conducted firstly, and then the method of SECT is used to achieve high-resolution TF representation. The result calculated by the proposed method is shown in Figure 6. It should be noted that the extracted ridge form of the optimized TF plane is shown in Figure 6b. Comparing Figure 5 with Figure 6b, it can be found that the proposed method has an obvious advantage in identifying the time-varying signal feature. Commonly, Rayleigh entropy is regarded as a significant criterion in reflecting the time-frequency aggregation. The smaller value of Rayleigh entropy always indicates a better performance of TF representation. The Rayleigh entropy calculated by different methods is listed in Table 1. The results demonstrate that the proposed method ISECT has a better performance in denoising and in sharpening TF ridges.

## 4. Experimental Data Analysis

### 4.1. Case 1: Data Analysis of a Fault Test Rig

Through the dynamic analysis, it can be seen that the natural frequency decreases and the characteristic frequency increases during the fault deterioration of rolling bearing. Therefore, the natural frequency of a vibration signal can reflect the severity of the rolling bearing fault. To prove the effectiveness of the proposed method, an experimental data analysis of a fault test rig is performed. To facilitate the rolling bearing analysis, the data analysis is used for the fault feature frequency identification. The experimental dataset was provided by the Machinery Failure Prevention Technology (MFPT) Society [29]. The test rig with a NICE bearing is performed to gather acceleration data for baseline conditions at 300 lbs of load. The structure parameters of the rolling bearing is described in Table 2.

**Figure 7:**
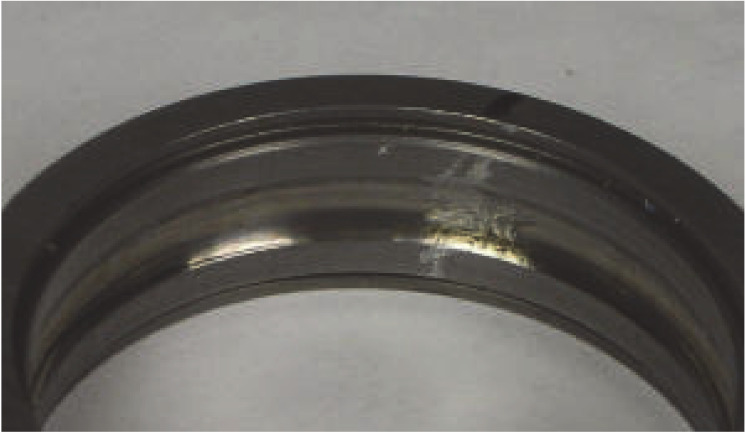
Outer race fault.

The data acquisition parameters are 25 Hz and 48,828 Hz for the input shaft rate and sample rate, respectively. The detailed calculation process can be found in [30]; the failure frequency of outer ring is f0=80 Hz. Figure 7 shows the outer ring fault.

**Figure 8:**
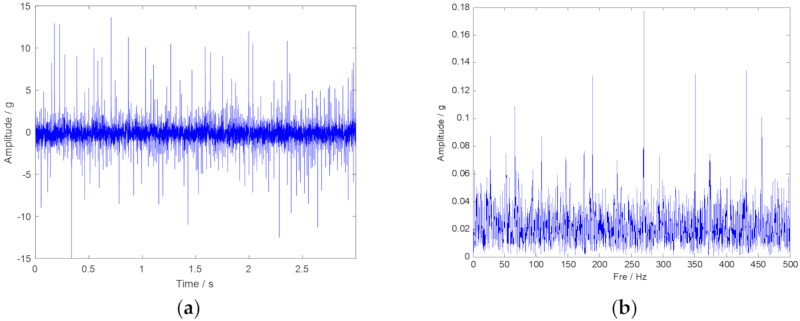
The time-domain and frequency-domain spectra of the original vibration signal. (**a**) Vibration signal shown in time-domain; (**b**) Vibration signal shown in frequency-domain.

**Figure 9:**
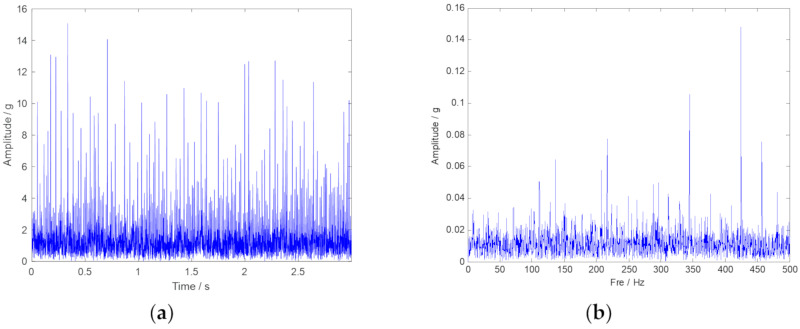
The time-domain and frequency-domain spectra of the envelope signal. (**a**) Envelope signal in time-domain; (**b**) Envelope signal in frequency-domain.

**Figure 10:**
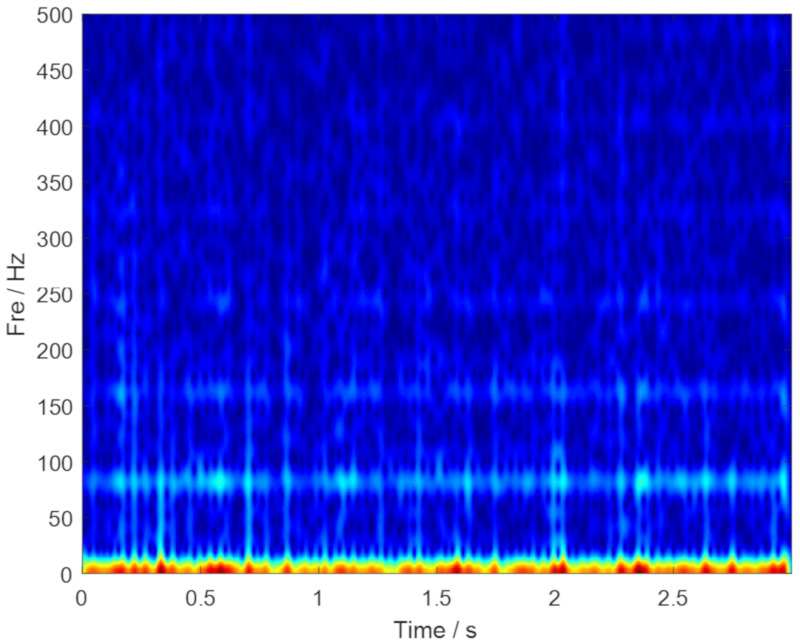
TF representations of the envelope signal.

The original vibration signal in the time-domain and the frequency-domain are plotted in Figure 8, respectively. The outer ring fault characteristic frequency in 80 Hz cannot been inspected in Figure 8b. Thus, the advanced signal processing method should be considered. Then, the envelope spectrum analysis is performed, and the corresponding result is plotted in Figure 9. Unfortunately, the fault characteristic frequency still cannot be inspected. TF representations of the envelope signal are plotted in Figure 10. Because of time-frequency ambiguity resulting from noise, we can only find the tendency of the outer ring fault characteristic frequency.

**Figure 11:**
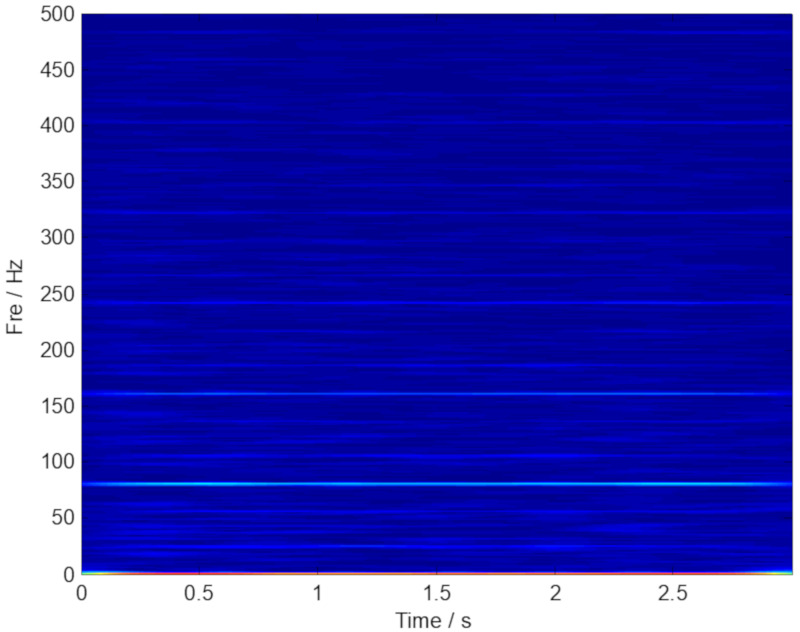
The result provided by ISECT method.

**Figure 12:**
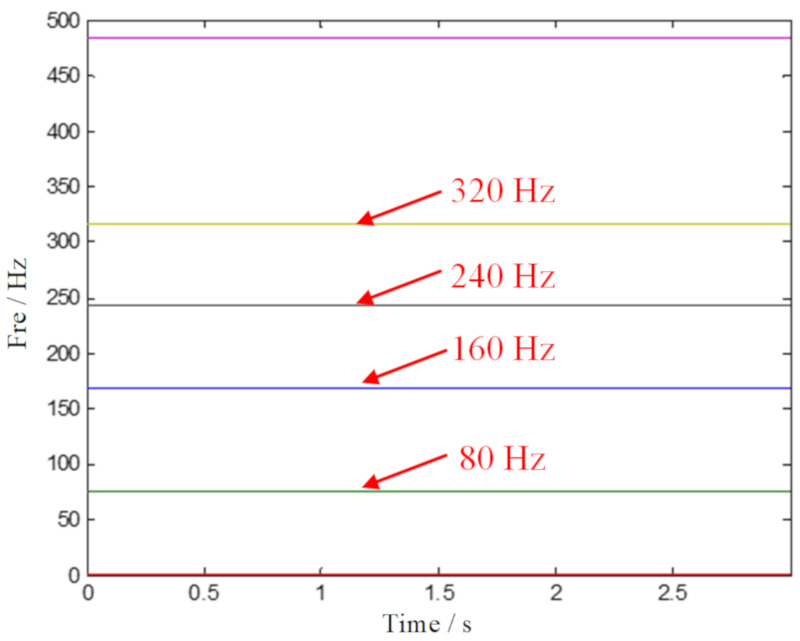
The multiple ridge identification by the proposed ISECT method.

The phenomenon of noise interference is the main problem to be solved in a TF analysis. One of the pre-processing means is to realize the signal denoising of complex multi-component signals. In this paper, the GMC denoising based on a convex optimization scheme is used to process the experimental data firstly. Subsequently, the SECT method is applied to the denoised signal, and the TF analysis result is shown in Figure 11. In order to clearly identify the features, a multiple ridge extraction to the SECT method is performed, and the result is plotted in Figure 12. According to Figure 12, we can clearly inspect the fault characteristic frequency of the outer ring and its harmonics. The diagnosis is consistent with the facts, and the superiority of the proposed ISECT method is demonstrated.

### 4.2. Case 2: Data Analysis of a Large Desulfurization Fan

**Figure 13:**
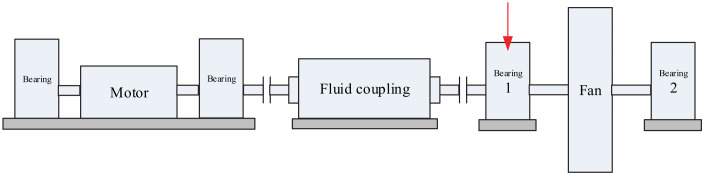
Schematic diagram of the large desulfurization fan structure.

The large desulfurization fan is a key equipment in a steel plant, and its main function is to remove dust and control environmental pollution. The structure diagram of the large desulfurization fan is shown in Figure 13. Once the equipment breaks down in the production process, the dust inside the converter cannot be removed, which not only pollutes the surrounding environment but also causes huge potential security problems. In order to ensure the normal operation of the large desulfurization fan, status monitoring and a diagnosis analysis on each part of the fan should be performed.

This paper mainly analyzes Bearing 1 on the left side of the fan. After testing, the bearing began to show abnormal vibration in September 2006 and was finally replaced on 1 February 2007. During this period, the CSI2130 vibration collector (Emerson, Missouri, TX, United States) was used to collect vibration acceleration signals in the vertical direction of the bearing. After the bearing replacement, a set of data was also collected for comparison on the new fault-free bearing. The collected data can be used to verify the performance of the proposed method in the extraction fault characteristics of the rolling bearing.

The input shaft speed of the hydraulic coupler is 850 r/min, and the output shaft speed is 740 r/min. The specific model of the rolling bearings on both sides of the fan is 22344CA (NSK, Kunshan City, China) , and the number of rolls is 13. Bearing operation parameters, data acquisition parameters, and bearing failure frequency are shown in Table 3. It should be noted that the outer ring fault feature frequency is calculated as f0=64 Hz.

**Figure 14:**
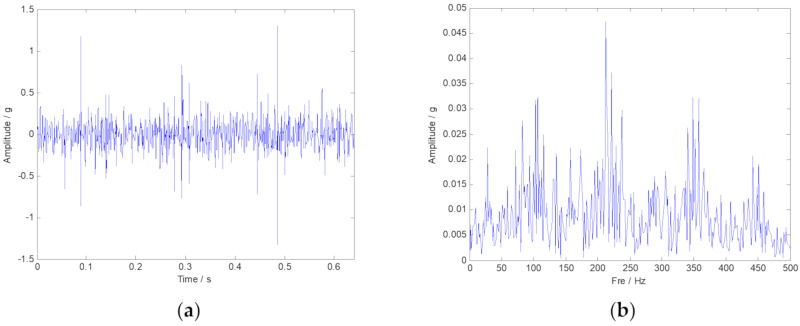
The time-domain and frequency-domain spectra of the original vibration signal. (**a**) Vibration signal shown in time-domain; (**b**) Vibration signal shown in frequency-domain.

**Figure 15:**
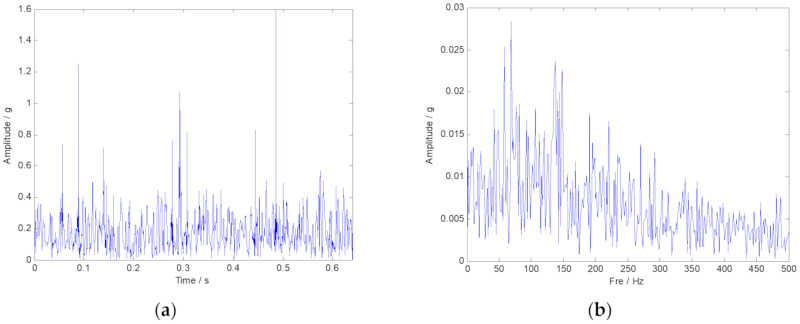
The result of the envelope spectrum analysis to the fan vibration signal. (**a**) Envelope signal shown in time-domain; (**b**) Envelope signal shown in frequency-domain.

**Figure 16:**
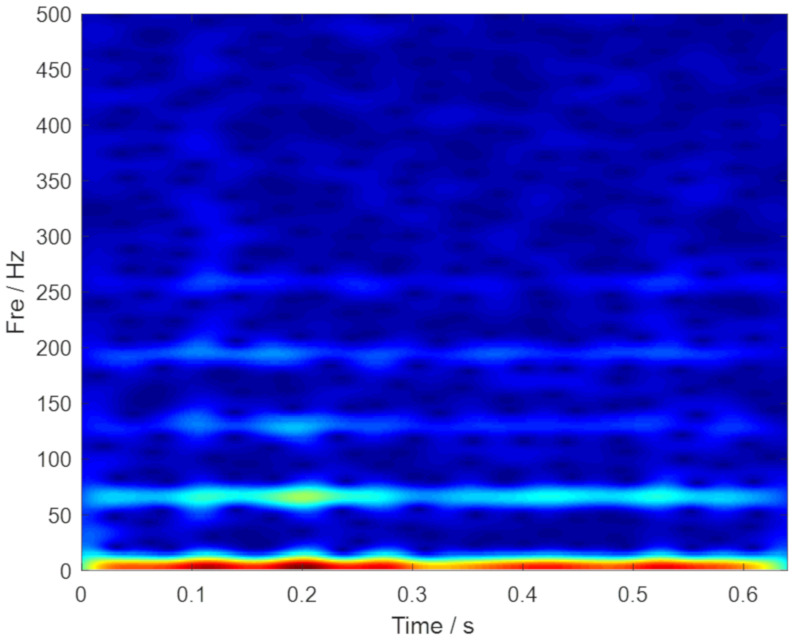
TF representations of the envelope signal of the fan.

The measured rolling bearing vibration signal of the large desulfurization fan is drawn in Figure 14. We still cannot identify the outer ring fault feature frequency in the frequency spectrum. Moreover, the noise interference components are easy to be inspected. Then, the envelope spectrum analysis was conducted, and the corresponding result is plotted in Figure 15b. Nevertheless, the fault characteristic components of the signal are masked by noise. Thus, the analysis results provided by the FFT and the envelope spectrum are unsatisfied. TF representations of the envelope signal of the large desulfurization fan are shown in Figure 16. We can hardly identify the phenomenon of the outer ring fault features from the fuzzy time-frequency plane.

**Figure 17:**
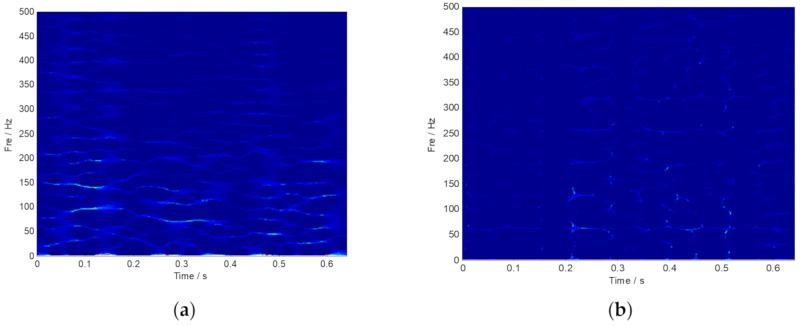
The result provided by the SST and SET methods. (**a**) TF representations generated by SST; (**b**) TF representations generated by SET.

Subsequently, the denoising operation is executed by the GMC penalty based on the convex optimization framework, which can improve the signal to noise ratio. Then, SST and SET, a novel TF analysis method, is applied to the vibration signal obtained by the proposed GMC denoising. The results provided by the SST and SET methods are plotted in Figure 17, respectively. However, the ridge curves related to the fault characteristic components are not obvious. Thus, the resolution of the TF representations of the fan vibration signal needs to be further improved.

**Figure 18:**
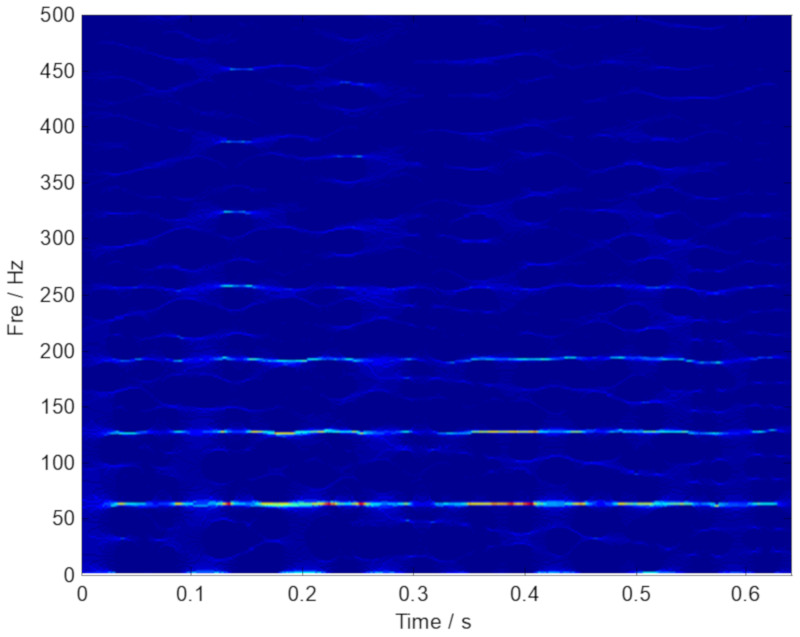
The result provided by the proposed ISECT method.

**Figure 19:**
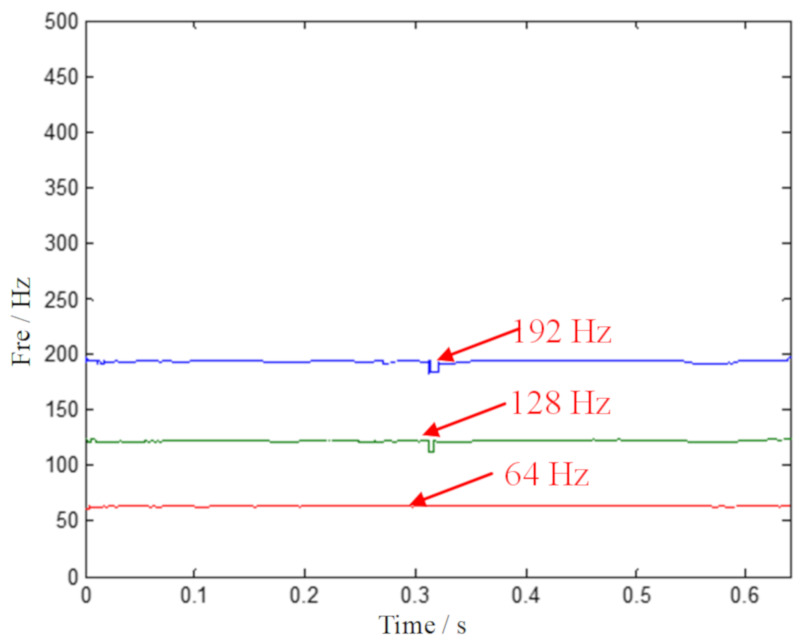
The multiple ridge identification by the proposed ISECT method.

Based on the GMC denoising scheme, SECT is put forward to achieve a concentrated TF plane. The denoised signal is processed by the SECT method, and the result is shown in Figure 18. The multiple ridge identification is plotted in Figure 19. This suggests that the interested signal component of the outer ring fault characteristic has a sharpened ridge, which is an obvious distinction from other extraneous components. This shows that the outer ring fault feature frequency and its multiplication can be easily extracted—that is to say, the effectiveness of the proposed method has been proven.

## 5. Conclusions

High-resolution time-frequency representations of multi-component signals are a key part in mechanical fault diagnosis. The noise is inevitable in the actual environment, resulting in large disturbances and unsatisfactory results. To overcome this shortcoming, the convex optimization with the GMC penalty function combined with SECT is introduced in this paper. The main contributions of this paper are summarized as follows: (1) The convex optimization algorithm with the GMC penalty function is used for signal denoising, which can enhance the readability of time-frequency representation; (2) the SECT algorithm is employed to achieve a concentrated time-frequency plane, which aims to accurately extract the ridge curves related to fault characteristic components; and (3) the proposed method ISECT can effectively extract the rolling bearing fault feature. Both the analysis results of the numerical simulation signal and the measured bearing failure data demonstrate that the proposed method has improved the time-frequency representations of multi-component signals. We recommend that the proposed approach can be applied to intelligent fault diagnosis.

## Figures and Tables

**Table 1:** Rayleigh entropy calculated by different methods.

Methods	STFT	SST	SET	SECT	Proposed ISECT
Rayleigh entropy	15.78	12.12	11.49	11.46	11.01

**Table 2:** The specific parameters of rolling bearing.

Roller Diameter	Pitch Diameter	Number of Elements	Contact Angle
0.235	1.245	8	0

**Table 3:** Experimental parameters and failure frequency.

Speed r/min	Rotational Frequency/Hz	Sampling Frequency/Hz	Sampling Time/s	Outer/Hz	Inner/Hz	Rolling Ball/Hz	Container/Hz
740	12.3	25600	0.64	64.1	96.2	34.4	5.1
